# Primary Cardiac Leiomyosarcoma Extending From Right Atrium to Inferior Vena Cava Without Metastasis: A Case Report

**DOI:** 10.7759/cureus.73826

**Published:** 2024-11-16

**Authors:** Jennifer Chong, Tanya Richvalsky, Sridhar Musuku, Sanjay Samy, Chikashi Nakai

**Affiliations:** 1 Cardiothoracic Surgery, Albany Medical Center, Albany, USA; 2 Anesthesiology, Albany Medical Center, Albany, USA

**Keywords:** cardiac thrombus, cardiac tumor in adults, leiomyosarcoma, metastasis, pathology

## Abstract

A 27-year-old female with a history of acute lymphoblastic leukemia in remission presented with chest pain, liver cirrhosis, and a thrombus in the hepatic vein on ultrasound. Further workup with computed tomography (CT) and magnetic resonance imaging (MRI) revealed a mass extending from the inferior vena cava to the right atrium, 3.4 x 3.4 x 7.0 cm in size. She underwent excision of the intracardiac mass with a full sternotomy and cardiopulmonary bypass successfully. Her postoperative course was uncomplicated. She was discharged home on postoperative day six. Subsequent histopathology confirmed high-grade leiomyosarcoma. Postoperative positron emission tomography CT showed no evidence of metastatic disease.

## Introduction

Cardiac tumors are relatively uncommon and can be either primary or metastatic in origin. Primary cardiac tumors are very rare, with the incidence being anywhere from 0.001-0.3% by autopsy reports [1]. Nearly 90% of all primary cardiac tumors are benign, with cardiac myxomas being the most common primary tumor. Other benign tumors include lipomas, papillary fibroelastomas, and fibromas. Malignant cardiac tumors account for only around 10% of all primary cardiac tumors [1]. Among the malignant primary tumors, angiosarcomas are most common, followed by rhabdomyosarcoma and fibrosarcoma. Leiomyosarcomas are very rare and originate from the left atrium, typically occurring in less than 1% of cases [1]. It is a mesenchymal tumor that occurs in smooth muscle cells, and therapy usually requires surgical resection if feasible, followed by radiation and chemotherapy. The prognosis of leiomyosarcoma is often very poor, with a mean survival of six months after diagnosis because of the rapidly growing and high rate of distant metastasis [2]. We present a young patient who has a past medical history of acute lymphoblastic leukemia (ALL) with a pathological diagnosis of leiomyosarcoma in the right atrium (RA) and inferior vena cava (IVC).

## Case presentation

**Figure 1:**
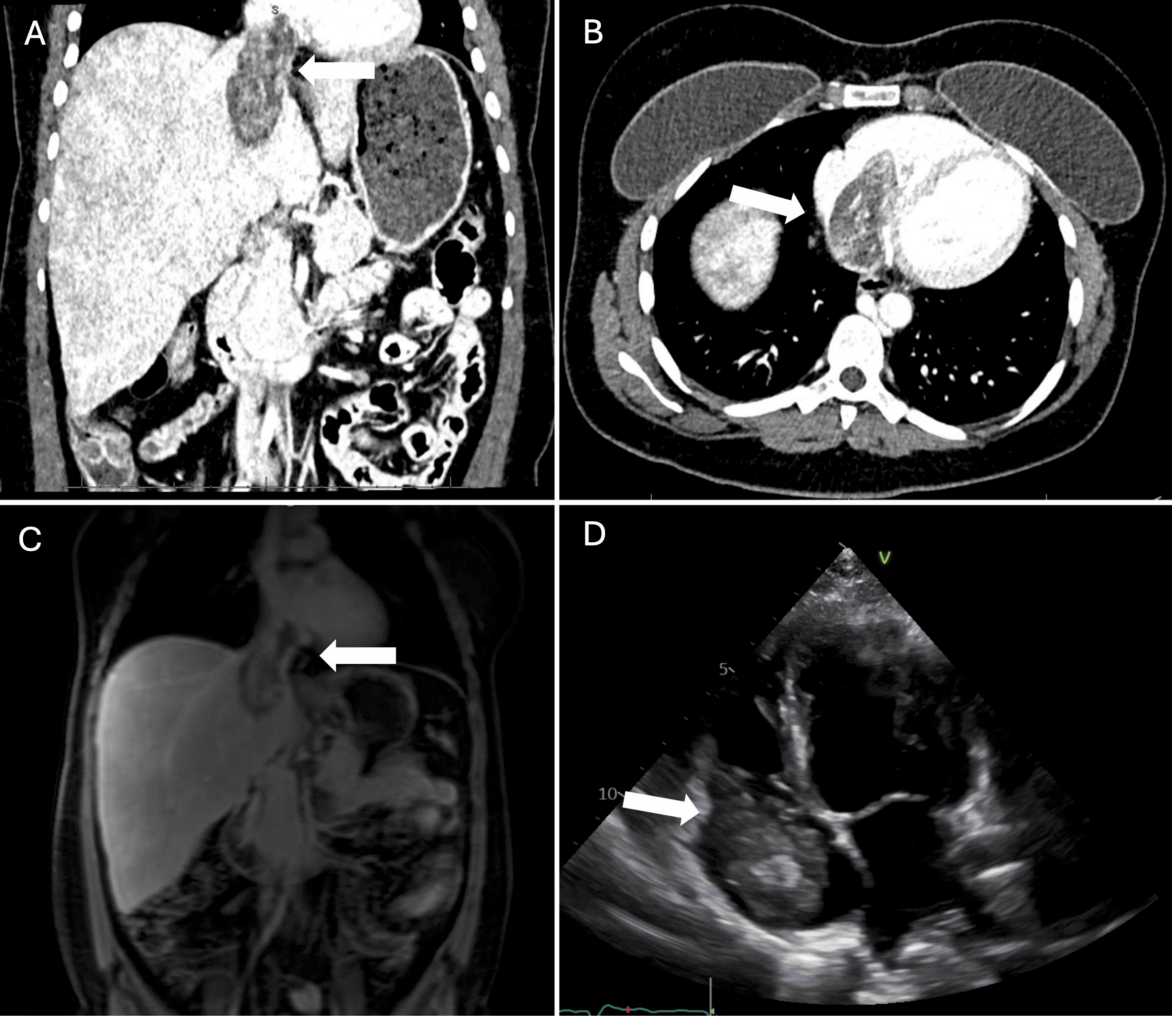
Preoperative CT, MRI, and TTE Figures 1A, 1B: CT abdomen with contrast: a mass in the IVC extending to the RA (white arrow). 1C: A mass 3.4 x 3.4 x 7.0 cm in size extending from the IVC to RA without evidence of focal hepatic lesion or renal mass (white arrow). 1D: TTE: A large mobile mass or thrombus in the RA that was prolapsed across the tricuspid valve into the right ventricle (white arrow)

**Figure 2:**
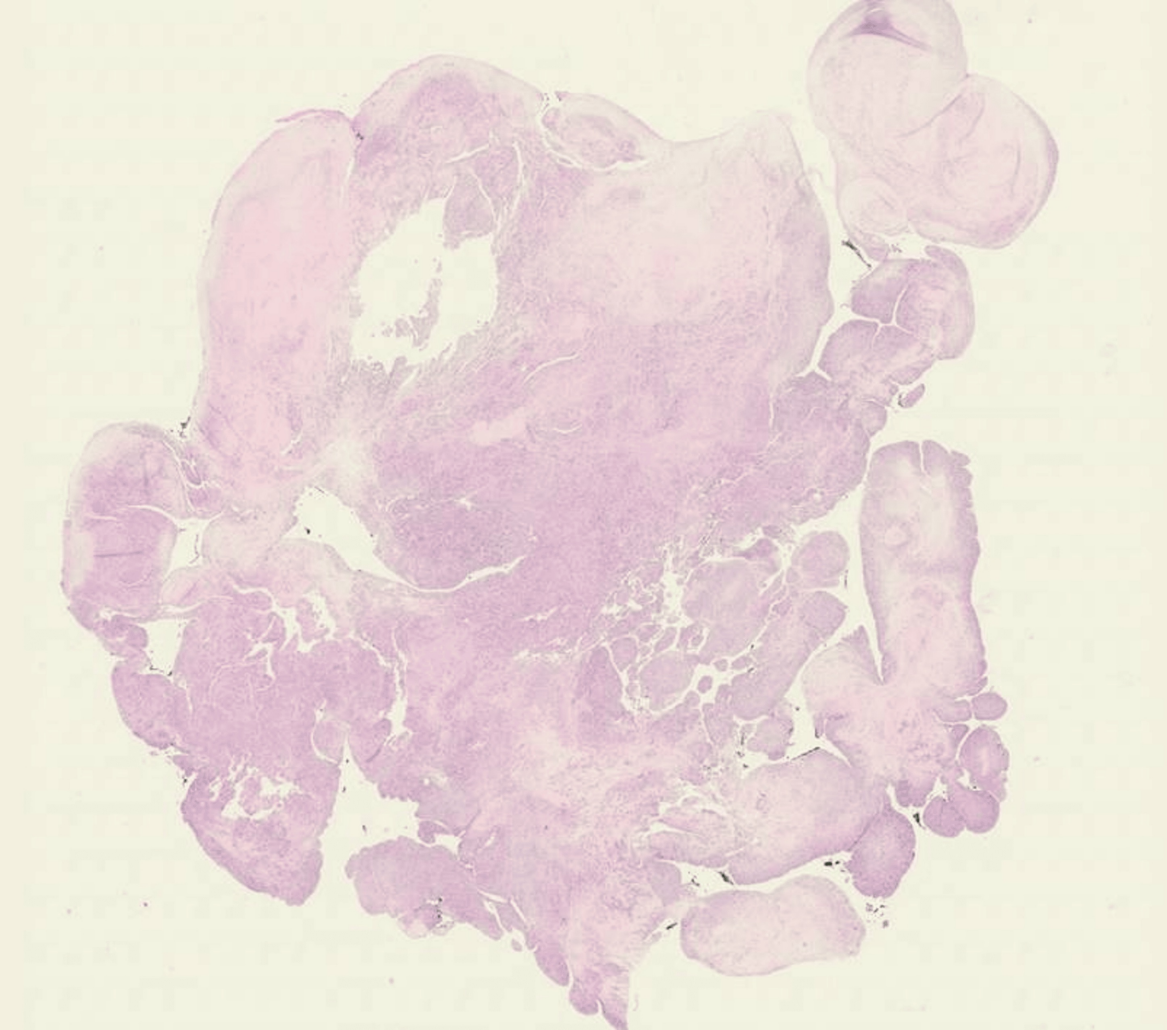
Histopathology of resected mass with hematoxylin and eosin (H&E) stain The presence of spindle cell sarcoma consistent with high-grade leiomyosarcoma

The patient is a 27-year-old female with a past medical history of ALL treated with chemotherapy, whole-body radiation, and an allogeneic stem cell transplant in her childhood, currently in remission. She presented with chest pain, shortness of breath, fatigue, and elevated liver function tests (Table 1) and was found to have liver cirrhosis and hepatic vein thrombosis on ultrasound. Oral anticoagulation was initiated for the thrombus. Computed tomography (CT) of the abdomen subsequently revealed a mass in the IVC extending to the RA (Figures 1A, 1B). She was admitted for further workup for the mass, and anticoagulation with continuous heparin was initiated. Magnetic resonance imaging (MRI) showed a mass 3.4 x 3.4 x 7.0 cm in size extending from the IVC to RA without evidence of focal hepatic lesion or renal mass (Figure 1C). A transthoracic echocardiogram (TTE) was performed to further delineate between the thrombus and cardiac tumor. TTE displayed a large mobile mass or thrombus in the RA that was prolapsed across the tricuspid valve into the right ventricle (Figure 1D). There were no specific findings in the imaging modalities to rule out thrombus or other diagnoses. While the next management was discussed in the multidisciplinary conference, she started to have presyncopal episodes with hypotension and systolic blood pressure down to 90 mmHg. The decision was made to excise the mass surgically for presyncope complicated by hypotension secondary to the IVC obstruction by the mass. Intraoperative findings in the transesophageal echocardiogram (TEE) were consistent with preoperative TTE. She underwent a full sternotomy, and cardiopulmonary bypass (CPB) was established with the arterial cannula in the ascending aorta and the venous cannula in the superior vena cava (SVC) and RA. After the cardiac arrest was achieved, the RA was opened with an incision down toward the IVC after the SVC venous cannula was snared down. The mass was noted in the body of RA adherents to IVC intima. She was placed on low flow with a pump sucker bypass to capture the venous blood into CPB without requiring circulatory arrest. It took approximately 30 minutes to extricate the mass from the RA and IVC successfully. On further inspection, there was a small atrial septal defect noted that was closed with a 4-0 polypropylene suture. CPB time and aortic cross-clamp time were 100 and 30 minutes, respectively. Her postoperative course was uncomplicated. Postoperative transthoracic echocardiogram showed no residual tumor in the RA. She was discharged home on postoperative day 6 with outpatient follow-up by the oncology service. Histopathology confirmed the presence of spindle cell sarcoma consistent with high-grade leiomyosarcoma (Figure 2). Postoperative positron emission tomography (PET) CT showed no evidence of metastatic disease. 

**Table 1:** Preoperative blood exam Elevated liver function test, including total bilirubin, alkaline phosphatase, and AST

Blood exam variables	Patient value	Reference range
WBC, 10*3/uL	9.5	4.1-9.3
RBC, 10*6/uL	5.69	4.00-5.10
Hemoglobin, g/dL	16.7	11.0-14.7
Hematocrit, %	50.2	33.0-44.0
Platelet, 10*3/uL	202	130-350
Creatinine, mg/dL	0.72	0.60-1.20
Urea Nitrogen, mg/dL	12	7-22
Albumin, gm/dL	5.1	3.5-5.2
Total bilirubin, mg/dL	2.7	0.1-1.2
Alkaline phosphatase, IU/L	145	30-115
AST, IU/L	50	5-45
ALT, IU/L	47	5-60

## Discussion

We experienced a case of primary leiomyosarcoma extending from the RA to IVC without evidence of metastasis. In our case, discerning whether the patient had a thrombus or a cardiac tumor posed a challenge, resulting in complexities in devising the treatment approach. Atrial tumors often present with non-specific symptoms such as arrhythmias, pericarditis, and dyspnea, as well as difficulties in regulating blood pressure. In the complex field of cardiac masses, a multimodality imaging approach is required to reach the diagnosis [3]. However, the clinical distinction between myxoma and sarcoma may be difficult [4]. Histopathological characterization remains the diagnostic gold standard in any resected cardiac masses to differentiate benign or malignant [3]. Surgical excision is the recommended approach for treating atrial tumors, while long-term anticoagulation is typically utilized to manage thrombi. In our case, anticoagulation and workup with imaging multimodality were performed as the initial management for the mass. However, the patient became symptomatic with presyncope complicated by hypotension during the workups. The decision was made to proceed with surgical resection, including histopathological diagnosis.

Leiomyosarcomas typically affect the uterus, abdomen, and retroperitoneum; sarcomas of the cardiac and vascular origin are exceedingly rare [5]. This type of neoplasm is difficult to diagnose since they are mainly asymptomatic in the early stages and can be confused with other heart pathologies. The left atrium is the most common site of origin, and leiomyosarcomas typically grow outwardly and have a high incidence of lung metastasis [6]. Surgical resection is considered for cases that present with hemodynamic compromise due to mass effect, increased risk of embolism, and the presence of cardiac arrhythmias [6]. The effectiveness of surgical resection depends on the neoplasia stage, the presence of metastases, and the patient's general condition [6]. The success of surgical treatment depends on the location of the tumor [7]. Leiomyosarcoma often recurs, and a repeated resection may be considered depending on the overall patient's status [7]. Regarding adjuvant therapy, leiomyosarcoma has low radiosensitivity, and often the risks of myocarditis due to radiation outweigh the benefits [3, 8]. The efficacy of chemotherapy is still unknown [5]. Leiomyosarcoma has a high rate of recurrence as well as reported cases of metastases after surgical resection. In our case, the patient did not have metastases at the time of diagnosis or post-surgical removal. However, the pathologic report did categorize the tumor as high grade with 50% necrosis and high mitotic activity, both representative of the high metastatic and proliferation rate of the tumor, often indicative of poor prognosis. In follow-up management, PET CT is useful for evaluating metastases. To remain in complete remission, a regular echocardiogram should be performed [9].

**Figure 3:**
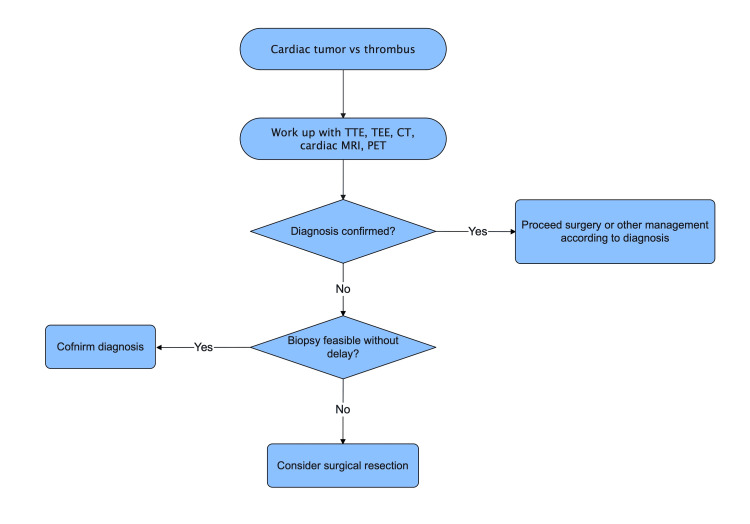
Management of cardiac mass from diagnosis to treatment Proceeding with surgical resection may be one of the options if the diagnosis is not confirmed with imaging modalities (TTE, TEE, CT, cardiac MRI, PET) or a biopsy is delayed or not feasible. Image credit: Chikashi Nakai

In our case, we seek to underscore the importance of rapid diagnosis and treatment for cardiac masses that may be commonly mistaken for benign pathology, such as an atrial myxoma or thrombus. Given her past medical history of ALL with radiation therapy, post-radiation sarcoma was one of the differential diagnoses. In general, radiation-induced tumors are high-grade and extremely rare in patients surviving more than 5 years after radiotherapy [10]. Rapid diagnosis and treatment of malignant cardiac tumors should allow the patient to avoid metastasis and obtain a better prognosis. Proceeding with surgical resection may be one of the options if the diagnosis is not confirmed with imaging modalities (TTE, TEE, CT, cardiac MRI, PET) or if biopsy is delayed or not feasible (Figure 3). Early diagnosis with multiple imaging modalities, biopsy, and adequate surgical resection remain the mainstays of leiomyosarcoma [9].

## Conclusions

Rapid diagnosis and treatment of malignant cardiac tumors should allow the patient to avoid metastasis and obtain a better prognosis. Proceeding with surgical resection may be one of the options if the diagnosis is not confirmed with imaging modalities or a biopsy is delayed.
